# Applying graphics processor units to Monte Carlo dose calculation in radiation therapy

**DOI:** 10.4103/0971-6203.62198

**Published:** 2010

**Authors:** M. Bakhtiari, H. Malhotra, M. D. Jones, V. Chaudhary, J. P. Walters, D. Nazareth

**Affiliations:** Department of Radiation Medicine, Roswell Park Cancer Institute, Buffalo, NY 14263, USA; 1 Department of Physics and Center for Computational Research, University at Buffalo, SUNY, Buffalo, NY 14260, USA; 2 Computer Science and Engineering, University at Buffalo, SUNY, Buffalo, NY 14260, USA

**Keywords:** Graphics processor unit, Monte Carlo

## Abstract

We investigate the potential in using of using a graphics processor unit (GPU) for Monte-Carlo (MC)-based radiation dose calculations. The percent depth dose (PDD) of photons in a medium with known absorption and scattering coefficients is computed using a MC simulation running on both a standard CPU and a GPU. We demonstrate that the GPU's capability for massive parallel processing provides a significant acceleration in the MC calculation, and offers a significant advantage for distributed stochastic simulations on a single computer. Harnessing this potential of GPUs will help in the early adoption of MC for routine planning in a clinical environment.

The use of Monte Carlo (MC) simulations is highly desirable for dose calculation in radiation therapy as part of treatment planning or verification. MC simulations represent the gold standard in radiation dose calculation since they include the real physics of the interactions of photons with materials.[1–4] Because of the complexity of the physics and atomic data of the interactions, solving the photon transport equations analytically is difficult or impossible. MC simulations track each photon through the medium by using random numbers to determine the occurrence of a particular interaction.[5] Since stochastic processes are involved, simulating a large number of photons is necessary to obtain reasonable statistical accuracy. This results in a computationally-expensive calculation. The standard CPU of a workstation or PC lacks the numerical performance for this task, and may require several hours for a typical MC dose calculation. In addition, manufacturers have not increased CPU clock speed recently, due to concerns about overheating and power consumption. One method of addressing this issue is to exploit the highly parallelizable nature of MC simulations, which permits calculations to be performed on a cluster of networked CPU's. For example, MC simulations performed at our institution employ a cluster of 1000 dual core Linux machines (2000 CPUs), maintained and administered by a local academic supercomputing facility. However, most clinics do not have access to such computational resources.

Fortunately, within the last several years, the programmable GPU has evolved into a powerful computing device, as illustrated by Figure 1.[6] With multiple cores driven by very high memory bandwidth, GPU's offer low-cost computational resources for both graphics and non-graphics processing.[7–9] Since it was designed primarily for graphics rendering, the GPU architecture devotes more transistors to data processing than to data caching and flow control. More specifically, the GPU is especially well suited to calculations which can be expressed as data-parallel computations and require a high ratio of arithmetic operations to memory operations.

**Figure 1:**
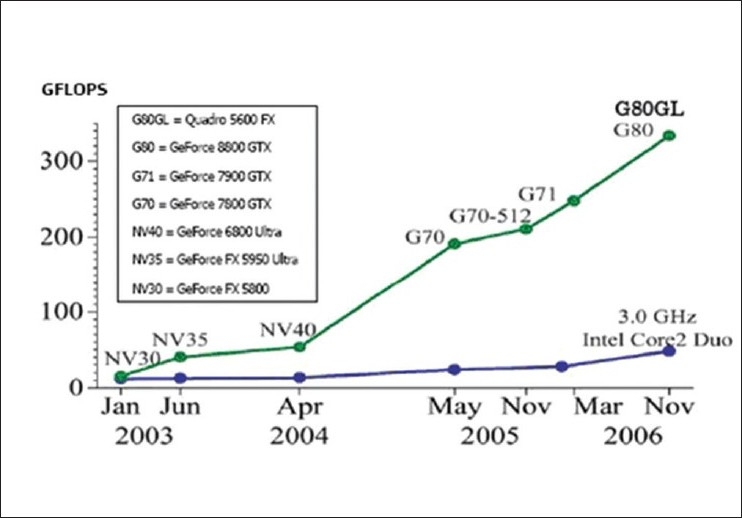
GPU versus CPU (source: www.nvidia.com)

We investigated the use of GPUs for MC dose calculations. Such an implementation would allow a physicist access to the computational resources of a supercomputer cluster in an inexpensive and portable machine. NVIDIA's (www.nvidia.com) recently-released GPU programming language, CUDA (Compute Unified Device Architecture) provided the necessary software tools.[9]

In order to compare the performances of GPU and CPU platforms for MC simulations, we developed a simple photon transport MC program. The code was implemented and run on both platforms, and the performances were compared. Therefore, the purpose of this technical note is not to describe a physically-accurate MC code, but rather to illustrate the potential advantages of GPU processing for this family of applications.

The simulation is performed in the following manner. A photon is incident on a semi-infinite isotropic medium, initially normal to the surface. The photon travels a distance of up to a value, s. During this process, the photon may undergo no interaction, or be scattered or absorbed. Since the medium is uniform, the absorption and scattering coefficients are constant throughout. No secondary-particle production is considered. If the photon is scattered, the new direction is selected randomly from a uniform distribution. When absorption occurs, the position of the absorption is recorded, and the simulation then proceeds with the next photon. All photons possess identical initial energy and are incident on the same point. A convolution technique can then be used to determine the dose distribution due to a beam profile of any shape. Figure 2 shows the dose distribution as a function of depth resulting from a simulation of 10^7^ histories. Note that since no secondary particle generation is considered, the curve achieves a maximum at zero depth, and the shape is different from a standard PDD.

**Figure 2:**
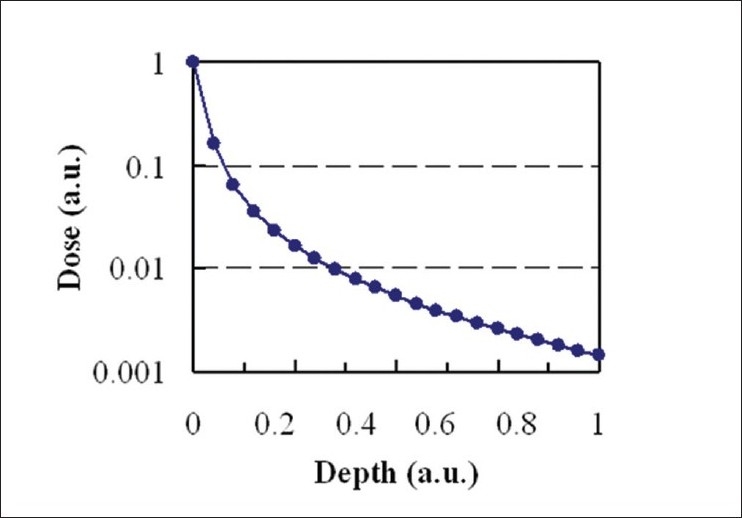
The influence obtained by the simple Monte Carlo simulation

We implemented the simulation on an Nvidia 8800 Ultra GPU using the CUDA language. The required random numbers were generated by the GPU, and the history of each photon was simulated using a separate thread. A total of 4096 concurrent threads were available. The program was then implented on a standard PC in the C++ language, and executed on the CPU for comparison with the GPU results comparison purposes.

The simulation times required for the CPU and GPU execution are shown in Figure 3. For a large number of histories, the GPU program is approximately 150 times faster, or equivalently, the GPU could simulate 150 times more histories than the CPU in a given amount of time

**Figure 3:**
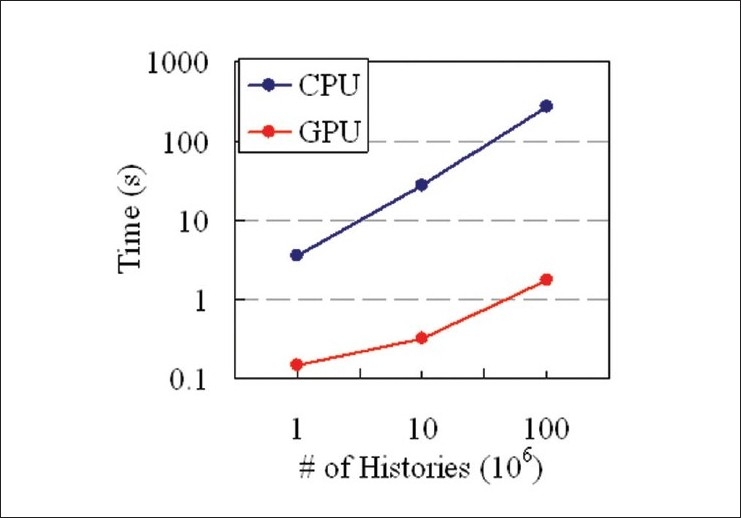
Comparing the CPU and GPU times for the Monte Carlo photon transport simulation

The potential was investigated of employing a GPU for MC simulation in radiation therapy. It was shown that the parallel data processing capabilities of the GPU can be harnessed to greatly increase the number of photon histories which may be simulated in a given time. The number was up to two orders of magnitude larger than that of the CPU simulation. This increase, using low-cost hardware and freely-available software tools, can provide enhanced accuracy of MC calculations, and make them feasible for clinical implementation.

This work was supported by an award from the Roswell Park Alliance Foundation. This research was supported in part by grant from NYSTAR. One of the authors, MB, would like to thank Dr. Matthew Podgorsak at Roswell Park Cancer Institute for supporting the work.
